# Glances at the Hospitals

**Published:** 1904-02-13

**Authors:** 


### GLANCES AT THE HOSPITALS.

### THE INGHAM INFIRMARY.

THE total income amounted to £3,550, and the expenditure to £3,455. This satisfactory state of the finances was in great part due to the workpeople themselves, who subscribed £1,579. The total number of in-patients was 517. The average daily number resident was 35, and the average stay in the infirmary was nearly 24 days. There is an analysis of the surgical cases treated during the year. We notice that ovariotomy was performed five times, all being successful. There were 36 amputations, with 2 deaths, and 9 cases of trephining the skull, 4 being successful, and 5 followed by death.

### THE KILMARNOCK INFIRMARY.

THE total income for the year was £5,081, and the expenditure exceeded this sum by £394; but it is pointed out that this adverse balance was caused by a large increase in the number of patients—in fact, the daily average number resident was 14 in excess of the number for the previous year. At the beginning of the year there were 133 patients in residence, and 1,195 were admitted during the year, making a total of 1,328 under treatment. Of these, 784 were discharged recovered, 217 improved, 51 not improved, 5 for other reasons, 102 died, and 156 remained in the wards. The average residence of fever cases was 47 days, of medical cases 27 days, and of surgical cases 31 days. In 300 fever cases the mortality was 7·6 per cent. In 309 medical cases the mortality was 9·5 per cent. In 503 surgical cases the mortality was 1 in 22·7. The greatest number resident in any one day was 169, and the smallest number was 88. The average cost per bed occupied was £45 14s., and the average cost per patient was £4 3s. 6d. The tables are compiled very carefully. There were 259 cases of scarlatina, and of these 194 recovered, 5 died, and 60 remained in the wards. Of 94 cases of enteric fever 56 recovered, 8 died, and 30 were under treatment. Of 19 cases of diphtheria 17 recovered and 2 died. Among the general medical diseases 26 cases of acute rheumatism gave the following results:—19 cured, 4 relieved, 1 died, and 2 remained under treatment. Of 48 cases of acute pneumonia

30 were cured, 3 improved, 2 not improved, 3 left before termination of treatment, and 10 died. A complete list of the operations performed during the year is given and results stated. There is also a table showing the causes of death, but we regret to notice that no anæsthetic table is given.

### THE ACCRINGTON VICTORIA HOSPITAL.

The total income was £1,466, and the expenditure was £1,455; so that the hospital is to be congratulated on living within its income. Mr. Richard Broughton has presented the institution with an *x*-rays apparatus, and it is proposed to enlarge the hospital for this purpose; an extension fund has been inaugurated, which has already reached the sum of £2,419, one subscriber, Mr. Appleby, having given £1,000. A further gift of £500 from Mr. Thomas Gordon has to be chronicled. There were 18 patients in the hospital at the beginning of the year. These with the admissions of the year made a total of 285. The average stay in the hospital was only 21 days, and the mortality rate was 4·5 per cent. The medical, surgical, and operation statistics are quite satisfactory in method of arrangement but we miss an anæsthetic table.

### MANCHESTER HOSPITAL FOR CONSUMPTION AND DISEASES OF THE THROAT.

The receipts from all sources were £3,757, and the expenditure was £4,062, leaving an adverse balance of £305. The Board, therefore, earnestly appeals for more funds to meet this deficit and place the hospital on a satisfactory basis; and the only way to obtain these desirable ends is to induce more people to subscribe annually. At present these subscriptions reach £1,343, and the total cost of the in-patients is £2,559, giving the average cost of each in-patient at 18s. 10d. a week. The out-patient department cost £1,502, but patients' payments reduced this by £935. During the year 1902, there were 378 patients admitted, and there were 49 in residence on the first of January. The average stay in the hospital was 43 days, but this calculation refers to all classes of patients. During the year 178 phthisical patients were discharged, and these show the following results:—32 were much improved, 116 were improved, 14 were stationary, 10 became worse, and 6 died. The average residence of consumptive patients was 82 days. The medical board state the pressure for beds remains unabated, and that many cases which are eminently fitted for admission have to be kept waiting for long periods, or have to be treated as out-patients. This is very deplorable, and the Manchester district is unfortunately no exception. Such a condition is found over the whole of England, and will be until the accommodation for patients able to pay sums under £1 a week is vastly increased. The medical board again state, and we are pleased to give their statement publicity, that sanatorium treatment can only be expected to effect a complete cure when the disease is taken under charge in the early stages. No confirmation is needed of this, but we find it amply borne out in the figures given on page 26 of the report. Of 91 cases in the first stage, 82 were improved, 4 were stationary, and 5 became worse. Of 62 in the second stage, 46 improved, 13 were stationary, and 3 became worse. Of 12 in the third stage, 5 improved, 4 were stationary, and 3 became worse.

### GUY'S HOSPITAL.

On January 1st this hospital contained 485 patients, and 7,706 were admitted during the year, making a total of 8,091 under treatment, being 2,537 in excess of the number treated during the year 1892. The average residence for medical cases was 37 days, and for surgical cases 17½ days. The mortality was 9·4 per cent. The out-patients reached the number of 124,963, and it is remarked that every section under this head showed an increase, more particularly the casualties and urgent cases. The governors have appealed to the public for a renovation and building fund, and for additional ordinary income, and the result of this appeal has been that, on the date of this report, a sum of £94,500 had been received or promised. The total expenditure for the year amounted to £108,201, and after supplementing the ordinary income by legacies and by donations, there was an adverse balance of £1,622, which was met by utilising cash balances, and by a transfer from the capital account. But the finances of the hospital are not so flourishing as this statement would lead one to believe, for at the end of the year previous a sum of £26,500 had been withdrawn from the re-endowment fund, and a further sum of £26,000 was owing to the bankers and others. Hence it is not without cause that the hospital appeals for funds to wipe off the debt, and to complete the scheme of renovation. The Henriette Raphael Nurses' Home was opened during the year by the Prince and Princess of Wales. The home provides complete living and sleeping accommodation for the nursing staff which now reaches a total of 236 persons. The average cost of each bed occupied was £105, which, compared with provincial hospitals, is literally enormous, and the average cost per patient was £6 6s. 1d. Several medical and surgical tables are given, but the information contained therein is useless for purposes of comparison as particular diseases are not mentioned.

### THE CARDIFF INFIRMARY.

The total income amounted to £10,535 and the total expenditure to £10,689. The workpeople's own contributions amounted to the respectable sum of £3,539. The number of in-patients was 1,878 and of these 822 were discharged cured, 622 were discharged relieved, 165 left for various reasons, 122 died, and 147 remained in the hospital on the date of the report. The average daily number resident was 132, and the average stay in the wards was nearly 26 days. The average cost per bed occupied was £63, and the average cost per head per day for provisions was 9d. We have often had to remark on the meagreness of the medical and surgical statistics supplied in some of these hospital reports, but the Cardiff Infirmary is about the only really important provincial hospital which supplies none at all.

### WEST CORNWALL INFIRMARY, PENZANCE.

The receipts were £994, and the expenditure was £853, leaving a credit balance of over £140, which the managers may well describe as "gratifying." It is proposed to rebuild the west wing of the hospital at a cost of about £1,350, and as the institution is now deriving one-third of its income from investments, it seems to us that this work might be safely undertaken; but as building estimates have a sad habit of insufficiency, we think the managers ought to have considerably more in hand than the £350 they speak of, or be prepared to withdraw more than £1,000 from their capital account. The total number of in-patients under treatment during the year was 118, and every one of these cases is detailed in the table, which shows not only the disease and the results of treatment, but the age, occupation, date of admission, date of discharge, and length of stay in the hospital. This is an admirable plan when reference has to be made to any individual patient, but it is not so good for purposes of comparison as the usual method. There is no anæsthetic table, an omission which we trust will be remedied in the next report; it is also to be hoped that there will be a statement showing the cost per patient, the cost per bed occupied, and the average period of each patient's residence in the hospital.

